# A Comparative Study in Metformin Tablet Quality Assessment: LC-MS and LC-MS/MS Method Quantification of N-Nitroso-Dimethylamine in the Presence of Dimethyl Formamide

**DOI:** 10.1155/ianc/5625153

**Published:** 2025-12-01

**Authors:** Gereziher Sibhat, Mariame A. Hassan, Indra K. Reddy, Mansoor A. Khan, Ziyaur Rahman

**Affiliations:** Irma Lerma Rangel College of Pharmacy, Texas A&M Health Science Center, Texas A&M University, College Station 77843, Texas, USA

**Keywords:** dimethyl formamide, mass spectrometry, metformin, N-nitroso-dimethylamine, quantitation of NDMA

## Abstract

N-nitroso-dimethylamine (NDMA, acceptable daily intake limit 96 ng/day), a probable human carcinogenic impurity, has been reported in metformin formulations. In some cases, it is difficult to accurately detect its presence due to suboptimal extraction technique(s), coelution of other probable human carcinogenic impurities such as dimethyl formamide (DMF) and/or ionization suppression in mass spectroscopic measurements. In this study, we provided a simple method to address DMF artifact using an affordable high-performance liquid chromatography coupled with a single-stage mass spectrometer. The results were further confirmed by dual-stage mass spectrometry. The two developed methods can simultaneously identify NDMA in the presence of DMF with good linear range from 10 to 100 ng/mL (*r*^2^ = 0.971) and 1 to 10 ng/mL (*r*^2^ = 0.994), respectively. The accuracy, expressed as % recovery, was close to 100% with overall precision (%RSD) < 8. The two methods were linear in the presence of 100 ng/mL DMF with *r*^2^ = 0.996 and 0.98, respectively. Five commercial formulations of metformin tablets showed traces of NDMA below the regulatory limit (0–12 ± 0.2 ng/tablet) but appreciable amounts of DMF (50–653.5 ng/tablet), still lower than the permissible daily exposure limit (8.8 mg/day) based on the maximum daily dose of metformin (5 tablets, 2500 mg). Both methods proved reliable and informative in the quality control of the NDMA content in metformin tablets and provided reciprocal verification of results.

## 1. Introduction

Drug recalls provide an effective protocol to minimize risk to patients from drug products, which fail to meet the minimum required quality and safety criteria after they are marketed. Out of the total number of the Food and Drug Administration (FDA) recalls on drugs in the last 10 years, 25%–37% are due to the presence of contaminants/impurities that pose potential risk to health in the final finished products [1, 2]. In general, the number of drug recalls increases with advancements in analytical methods as more undesired contaminants and degradation products are being discovered [3]. In 2018, carcinogenic nitrosamine contaminants/impurities were discovered in drug products, and prompt action was taken worldwide [4–7].

Since then, the extending list of susceptible pharmaceutical products (the cohort of concern) has been the focus of attention of both the regulatory agencies and the research community due to the challenges in eliminating/controlling these compounds [8]. Several reports indicated that the nitrosamine impurity NDMA is found to be mutagenic in experimental animals [9]. The presence of very low level of residual amines from the active pharmaceutical agent and a nitrite sourced from any molecule can result in the formation of NDMA in pharmaceutical preparations [10, 11]. As a result, the ICH guideline recommends a thorough assessment and control of mutagenic impurities in pharmaceuticals to limit potential carcinogenic risk [12].

Numerous analytical methods have been developed in an effort to accurately detect and quantify nitrosamines in finished pharmaceutical products [13]. Nitrosamines can be broadly classified into small-molecule nitrosamines such as N-nitroso-dimethylamine (NDMA) [14], N-nitroso-diethylamine (NDEA), and N-nitrosamine drug substance-related impurities (NDSRIs). NDSRIs are a class of nitrosamines that are structurally similar and unique to each API [15]. Small-molecule nitrosamines present the most challenging group in their analytical detection and quantification due to their small molecular size, good solubility in a wide range of common solvents such as water, methanol, and diethyl ether, semi-volatility, ease of formation under the conditions of extraction, and the low abundance relative to the dug substance(s) and/or excipients. Many techniques have been proposed to efficiently extract and concentrate them prior to analysis including liquid–liquid extraction [16, 17], salting-out liquid–liquid extraction [18], dispersant-first liquid–liquid extraction [19], solid phase extraction [20–22], precipitation [23], microextraction [24, 25], besides direct extraction in water or methanol or their combinations. Despite these techniques, there still is a caveat in the analyses when other inevitable impurities are present together with nitrosamines, for example, dimethyl formamide (DMF), nitrites, pyrrolidone, and so on [26]. Not only do these impurities interfere with nitrosamine peaks but also may induce a positive loop favoring the formation of nitrosamines. In 2020, a report submitted to the FDA claimed that 42% of 38 marketed products of metformin failed to meet safety requirements for their above-threshold NDMA content (acceptable intake; AI is 96 ng/day) [27]. Interestingly, further investigation by the FDA halved this percentage to 21% of the products. The discrepant results were attributed to the interference by DMF [28, 29].

**Figure 1:**
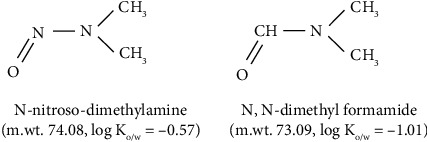
The structure of N-nitroso-dimethylamine (NDMA) and N, N-dimethyl formamide (DMF). m.wt = nominal molecular mass based on the relative abundance of isotopes approximated to 2 decimals. Log Ko/w is the logarithm of the partition coefficient (K) between oil (o) and water (w).

DMF is a commonly used solvent in the pharmaceutical industry [30]. It is involved in the synthesis of many drug substances (“Method for preparation of high purity and high yield metformin hydrochloride by two-component solvent. https://patents.google.com/patent/CN104829495A/en”; [31]) and its residual amounts in the finished pharmaceutical products are capped by ICH guidelines to 8.8 mg/day (880 ng/mg) due to its toxicity International Council for Harmonization of Technical Requirements for Pharmaceuticals for Human Use. Impurities: Guideline for residual solvents Q3C [32, 33]. DMF, like NDMA, is classified as a Group 2A probable human carcinogen by the International Agency for Research on Cancer (IARC); though for DMF, this occurred relatively recently in 2018 [34]. The reclassification of DMF occurred a year after the most recent, 2017, FDA review of regulatory limits set for DMF, and request to revisit the limits on DMF given IARC's reclassification is the focus of a still pending FDA Citizen Petition filed in 2019 (2019). It can also be hydrolyzed under certain conditions to produce dimethylamine, which can react to form NDMA or NDSRI in the presence of a nitrosating agent(s). Thus, DMF has three undesirable attributes to the pharmaceutical industry such as (1) a health hazard [35], (2) a precursor to nitrosamines [36], and (3) a coeluting molecule owing to its chemical and physical similarity to NDMA. DMF and NDMA molecular masses are very close (namely, m/z is 74.09 and 75.08 in positive ionization mode, respectively) (Figure 1). It has been postulated that if DMF is present in sufficiently high amounts (but not necessarily violating current regulatory levels), it can interfere with the acquisition and integration of NDMA peaks resulting in overestimation of the latter content [29]. In 2023, the European Union has restricted the use of DMF in many industries for safety reasons Commission Regulation [37]. All these issues should incentivize pharmaceutical companies to find alternatives to the use of DMF [38]. Until this comes fully in effect, DMF remains to present a hazard and a potential analytical artifact in judging the level of carcinogenic impurities in important drug products, such as metformin.

In this study, a rapid and less expensive method for screening and detecting NDMA in the presence of DMF by ultraperformance liquid chromatography (UPLC) coupled with single-stage mass spectrometry (MS) was provided. The results were further compared to the higher sensitivity more expensive tandem mass spectroscopy (MS/MS) equipment. Both methods are simple and reliable in detecting both NDMA and DMF simultaneously. The utility of liquid chromatography coupled with MS has been demonstrated across diverse fields, from the characterization of complex natural products in plant extracts to the sensitive detection of trace-level impurities in pharmaceuticals [Ref]. This technique's high mass accuracy and resolution are critical for confident analyte identification, a principle that is equally vital for distinguishing between closely related impurities like NDMA and DMF in drug products [39]. Five commercial metformin products suspected to contain high amounts of NDMA were selected for the study. Metformin is a first-line medication for type 2 diabetes with a well-established safety profile. It is among the top 20-prescribed medications with a market size of $4.17 billion in 2024 [40, 41] Its therapeutic potential is continually being explored, with recent research indicating benefits beyond glycemic control, such as promoting peripheral nerve regeneration by inducing M2 macrophage polarization via the AMPK/PGC-1α/PPAR-γ pathway [42]. This expanding therapeutic promise underscores the critical importance of ensuring the drug's safety from carcinogenic impurities like NDMA, which could jeopardize patient safety and undermine its clinical value. Also, some insights into the extraction techniques and sample preparation from the literature and the optimization studies are discussed.

## 2. Materials and Methods

### 2.1. Materials

LC-MS-grade formic acid, methanol, dichloromethane (DCM), acetonitrile (ACN), NDMA (Lot # 0006541941), and DMF (Lot # ZG396677) were purchased from Fisher Chemicals, Asheville, NC. In-house water (18 MΩ.cm, Millipore Milli-Q Gradient A-10 water purification system) was used in the study. Commercial metformin tablets were obtained from licensed wholesalers.

### 2.2. Methods

#### 2.2.1. Calibration Standards

A 1000 ng/mL stock solution of NDMA was prepared in methanol from which a working standard of 100 ng/mL was prepared in the same sample solvent depending on the method. This latter solution was used in the preparation of the calibrators and the quality control standards. Calibration standards for UPLC-MS were from 10 to 100 ng/mL in 2% formic acid in water, a range to cover from 0.1*x* to 1*x*, where *x* is the AI of NDMA of 96 ng/day [23]. Owing to the higher sensitivity of the LC-MS/MS, the range was modified to 1–10 ng/mL in water. A similar 1000 ng/mL stock solution of DMF was prepared in methanol. DMF was spiked at different concentrations, namely, 1, 10, 50, 100, and 150 ng/mL to confirm NDMA peak separation.

#### 2.2.2. UPLC-MS (LC-MS)

A UPLC (Acquity H-Class Plus System, Waters Corporation, Milford, MA) consisting of a QDa mass detector, a PDA detector, a temperature-controlled column compartment, and an autosampler with a quaternary solvent manager was used. A reverse-phase Kinetex 2.6 μm PFP, 4.6 × 100 mm column (Phenomenex, Torrance, CA) with a C18 UPLC SecurityGaurd guard column (Phenomenex, Torrance, CA) was connected to the system. The column temperature was kept at 40°C, and the samples were kept at 10°C. The mobile phase composition was water (mobile phase A) and methanol (mobile phase B) with formic acid added in both phases at a concentration of 0.1%. The elution gradient was 0.0–2.4 min 65% mobile phase A and 35% mobile phase B, 2.41–3.0 min 80% mobile phase A and 20% mobile phase B, and 3.01–5 min 50% each of the phases. The flow rate was 0.4 mL/min. The injection volume was 50 μL. The QDA mass detector was equipped with a heated electrospray ionization source operated in a positive ion mode with a nitrogen gas flow at 100 psi and gain equal to 1. Differential mass settings are listed in Table 1. NDMA and DMF detection were at *m*/*z* 75.0 and *m*/*z* 74.0, respectively. Empower 3 software was used for tuning parameters, data acquisition, and processing.

#### 2.2.3. UPLC-Mass-MS (LC-MS/MS)

LC-MS analysis was performed on an Acquity UPLC H-Class Plus System coupled to a Xevo TQD triple quadrupole mass spectrometer (Waters, Milford, MA). Chromatographic separations were achieved on a reverse-phase Kinetex 2.6 μm PFP, 4.6 × 100 mm column (Phenomenex, Torrance, CA) with a C18 UPLC SecurityGaurd guard column (Phenomenex, Torrance, CA) set at 40°C. The mobile phase was composed of water (mobile phase A) and methanol (mobile phase B) with formic acid added in both phases at a concentration of 0.1% and pumped at a flow rate of 0.4 mL/min for 7 min. The elution gradient was optimized to be 0.0–2 min 98% mobile phase A and 2% mobile phase B, 2.1–5 min 10% mobile phase A and 90% mobile phase B, and 5.01–7 min 98% mobile phase A and 2% mobile phase B. The sample tray temperature was set at 10°C. The injection volume was 50 μL. The needle was set to pre- and postwashes with methanol at 10 s each. The instrument was operating in a positive ion mode. The optimized settings are given in Table 2. MassLynx and TargetLynx software were used in instrumental control and data processing, respectively.

#### 2.2.4. Method Validation

Method validation was carried out in accordance with the International Council for Harmonization (ICH) and The United States Pharmacopeia (USP) [16, 43]. A stock solution (1000 g/mL) and working standard solution (100 ng/mL) of NDMA were prepared in methanol. The specificity of the method was assessed by injecting the appropriate blank solvent (DCM, methanol, 50% methanol in water, water), NDMA (10 and 100 ng/mL for LC-MS, and 1 and 10 ng/mL for LC-MS/MS), and DMF (100 ng/mL). The accuracy of the method expressed as the percent recovery was determined by injecting a known amount of NDMA at four concentration levels (*n* = 6), namely, 10, 40, 70, and 100 ng/mL and 1, 3, 5, and 10 ng/mL for LC-MS and LC-MS/MS, respectively, corresponding to LOQ, low, medium, and high NDMA concentrations in the calibration curves. Precision of the methods was also evaluated at the same concentration levels. The methods were validated over 3 days. Other parameters such as limit of detection (LOD), limit of quantification (LOQ), linearity (correlation coefficient *r*^2^ ≥ 0.98) as well as accuracy (70%–130%) and precision (RSD < 25%) were also calculated. A physical mixture (PM) composed of pharmaceutical excipients and metformin (500 mg) was used to test the matrix interference. The PM contained hypromellose, povidone, polyethylene glycol, corn starch, and magnesium stearate (200, 100, 90, 100, and 10 mg/unit dose) based on the composition of the selected commercial metformin products. The chromatograms of the PM samples spiked with 100 ng/mL of NDMA and 10 ng/mL for LC-MS and LC-MS/MS, respectively, were used to look for any interfering peak(s) in the region of NDMA and DMF peaks.

#### 2.2.5. NDMA Extraction

Preliminary work aiming to optimize extraction procedures involved the screening of different solvents such as water, ethanol, methanol, DCM, ether, cyclohexane, and hydroalcoholic mixtures at various concentrations. Different solvent volumes (3, 5, and 10 mL) were also tested. The extraction time was varied from 0.5 to 48 h. In all scenarios, direct supernatant injection results were compared to modified techniques intended to purify and concentrate the extract (e.g., drying followed by reconstitution or liquid–liquid extraction). In these studies, spiked PM samples were used. Spiking was always carried out at the lowest and highest NDMA concentrations, namely, 10 and 100 ng/mL for LC-MS and 1–10 ng/mL for LC-MS/MS and 100 ng/mL of DMF as appropriate.

In the final direct extraction method, three tablets were crushed individually and suspended in 10 mL of 50% methanol in water in a 50 mL falcon tubes. The resulting mixture was sonicated for 2 min and then kept in a shaking water bath (100 rpm, 25°C) for 1 h. Approximately 1.5 mL volume from each solution was transferred into a 2 mL microtube and centrifuged for 30 min at 10,000 rpm. Finally, 1 mL of the supernatant was transferred into an HPLC vial for analysis.

**Figure 2:**
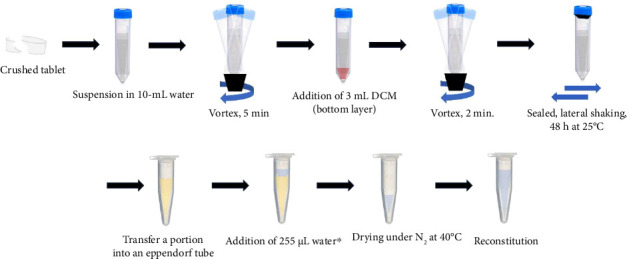
Summary of the liquid–liquid extraction method. ^∗^Water: 2% formic acid in water was used for the LC-MS method, while only water was used for the LC-MS/MS method.

In the final liquid–liquid extraction method, three tablets were crushed individually and suspended in 10 mL of water in 50 mL falcon tubes. The samples were vortexed for 5 min, 3 mL of DCM was added, and the mixtures were vortexed for an additional 2 min. The two liquid phases were immiscible with the DCM as the bottom layer. The samples were carefully sealed and kept on a lateral shaker for 48 h at 25°C. The bottom layer was then carefully collected in clean tubes. For the single-stage mass analysis, the whole DCM layer was evaporated after adding 255 μL of 2% formic acid in water. For the tandem mass analysis, 200 μL portions of the DCM layer were aliquoted for evaporation after adding 50 μL of water to each portion. Evaporation was performed in a water bath nitrogen evaporator with a nitrogen gas pressure set at about 5–8 psi and a temperature at 40°C. The remaining aqueous layer was made to 300 or 200 μL by 2% formic acid in water or water, respectively. The samples were further centrifuged at 10,000 rpm for 15 min at 4°C to remove any undissolved particles prior to injection.  Figure 2 provides an illustrative diagram of the extraction steps.

## 3. Results and Discussion

### 3.1. Development of LC-MS Method to Resolve the DMF Peak

**Figure 3:**
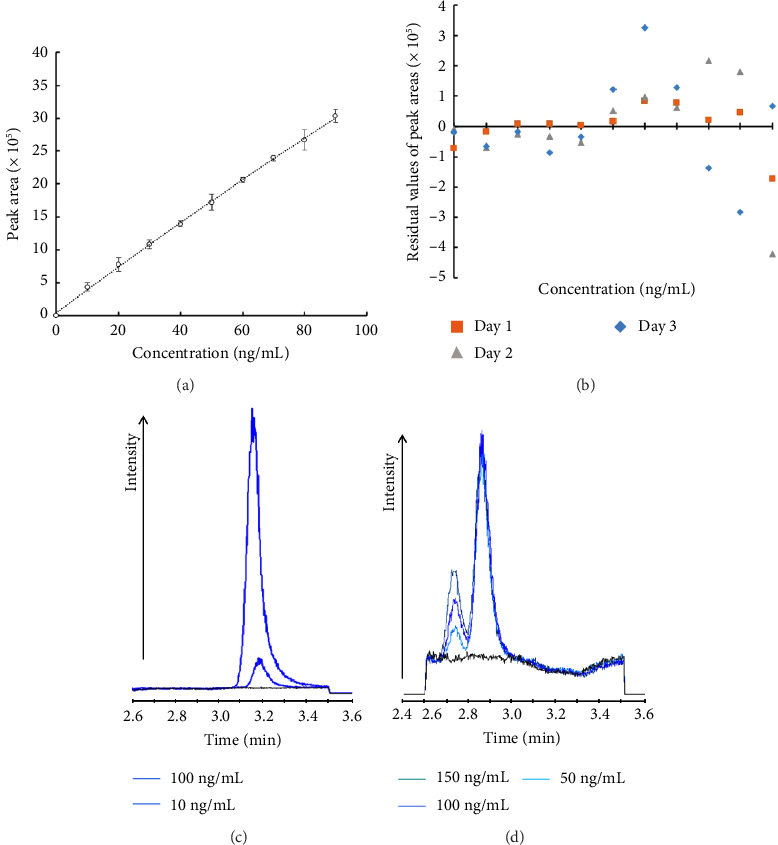
LC-MS method for the simultaneous detection of NDMA and DMF. (a) The calibration curve of NDMA. (b) The residual plot. (c) The chromatogram of NDMA (m/z = 75) at 10 and 100 ng/mL. (d) The chromatogram of DMF (m/z = 74) at three different concentrations (50, 100, and 150 ng/mL).

The first goal was to choose a stationary phase that achieves higher retention of polar analytes and to prolong the retention time (RT). This could be achieved through using columns with different hydrophobicities. Three columns with different hydrophobicities according to the hydrophobicity subtraction model (hydrophobic interaction H term) were selected [44, 45]. These were a Phenomenex Kinetex PFP (2.6  μm, 4.6 × 100 mm), a Waters Xselect HSS T3 (1.8 μm, 2.1 × 100 mm), and a Waters Xselect HSS (2.5 μm, 2.1 × 100 mm) with *H* = 0.68, 0.94 and 1.02, respectively. The ACN in the mobile phase was replaced with methanol that has less eluting strength [46]. A 0.1% formic acid was added to both mobile phases to enhance the ionization [47, 48]. The best separation of analytes was achieved using a Kinetex PFP column (NDMA RT was around 3 min). The next goal was to achieve sufficiently good resolution between NDMA and DMF peaks. The key principle underlying this maneuver is the differential hydrophilicities of both substances. The reported partition coefficient of both molecules indicates that DMF has more affinity to the aqueous phase compared to NDMA (o/w partition ratio is 1:10.2 parts vs. 1:3.7 parts for DMF and NDMA, respectively) [14, 49]. This means that if both compounds are retained on a hydrophobic column (hence reversed phase chromatography), the DMF peak can be eluted first provided that the polarity of the mobile phase is carefully fine-tuned. Systematically planned different proportions of water and methanol, both containing 0.1% formic acid, were used in an isocratic elution mode for screening purposes. The preliminary data showed that the ratios of the mobile phases A: B between 50:50% and 70:30% could separate the peaks of DMF and NDMA. This range was further narrowed down to achieve the best resolution. The optimized gradient started with A: B at 65:35% to induce the maximum peak resolution, then ramped up to 80:20% to flush out the column and avoid carryover to the next sample, and finally going down to 50:50% for reconditioning before the next injection. The systematic optimization of the chromatographic gradient was crucial not only for achieving baseline resolution between NDMA and DMF but also for ensuring high-quality peak shapes for accurate integration and quantification. The integrity of peak shape is a critical factor in metabolomics and impurity analysis, as suboptimal peak-picking can significantly impact data accuracy and reproducibility [Ref]. The final method, utilizing a Kinetex PFP column with a water/methanol gradient containing 0.1% formic acid, produced sharp and symmetrical peaks for both analytes, which facilitated reliable detection and minimized integration errors [50]. The MS settings, namely, the capillary and cone voltages, were also differentially tuned to achieve the best peak intensities (Table 1). The method showed good linearity (*r*^2^ = 0.971) and repeatability over the workdays for NDMA (Figures 3(a) and 3(b)). The accuracy expressed as % recovery was close to 100% for most samples except for the lowest concentration (82.6% ± 10.3%, 97.7% ± 2.3%, 104.4% ± 2.3%, and 94.5% ± 8.1% at 10, 40, 70, and 100 ng/mL, respectively). Further, precision of the method was evaluated with respect to % RSD of the QC samples, which was found to be 12.4% ± 10.4%, 1.5% ± 2.2%, 2.0% ± 2.2%, and 4.1% ± 5.8%). The overall average precision was < 5%, indicating that the method was precise in quantifying NDMA. The summary of the method validation is given in Table 3. The recovery after extraction of NDMA from the PM samples spiked with 10 and 100 ng/mL was 70.7% ± 15.9% and 95.5% ± 10.7%, respectively. The method was linear in the presence of 100 ng/mL of DMF with *r*^2^ = 0.996. DMF peaks were well-resolved from NDMA at different concentrations—combinations of NDMA and DMF as shown in Figures 3(c) and 3(d) (resolution = 1). The recovery of NDMA at 10, 40, 70, and 100 ng/mL in the presence of 100 ng/mL of NDMA was 108.5% ± 13.2%, 111.2% ± 0.6%, 110.0% ± 1.2%, and 97.1% ± 0.2%, respectively.

### 3.2. Development of Confirmatory LC-MS/MS Method

**Figure 4:**
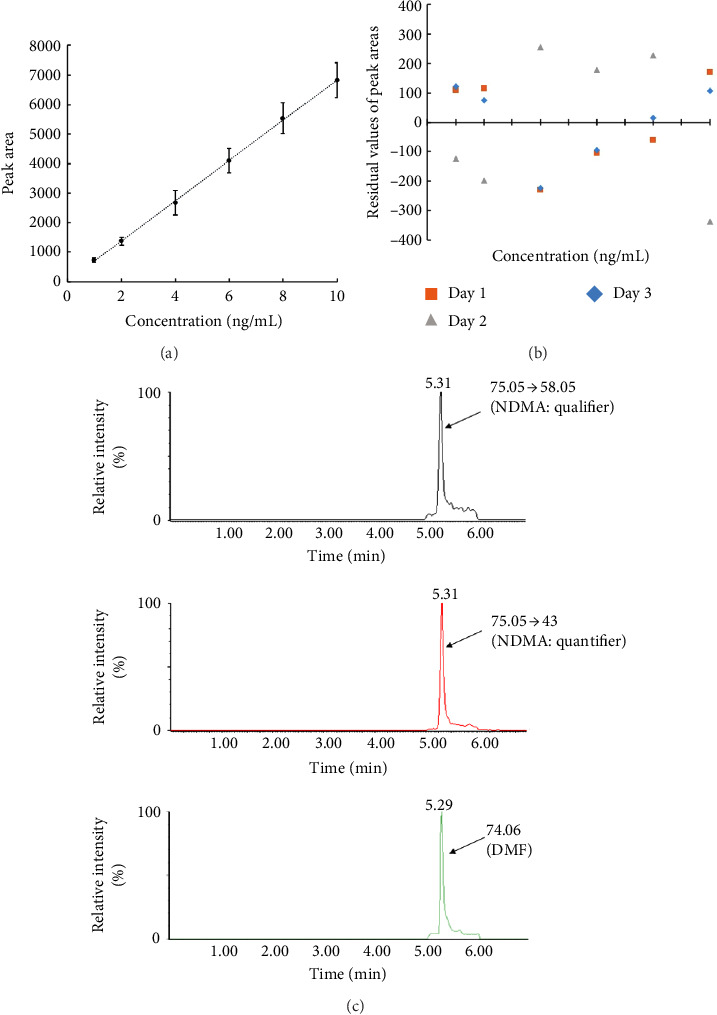
LC-MS/MS method for the simultaneous detection of NDMA and DMF. (a) The calibration curve of NDMA. (b) The residual plot. (c) The chromatogram of NDMA ion products (m/z = 75.05 ⟶ 58.05, qualifier ion, top, and m/z = 75.05 ⟶ 43, quantifier ion, middle) and DMF (m/z = 74.06, bottom).

The previous method was applied to a Water Acquity UHPLC coupled to a triple quadrupole mass spectrometer (LC-MS/MS) with slight modification. A lower range of NDMA standard solutions from 1 to 10 ng/mL was used. Initially, the three columns Waters Xselect HSS T3 (1.8 μm, 2.1 × 100 mm), Xselect HSS (2.5  μm, 2.1 × 100 mm), and Phenomenex Kinetex PFP (2.6 μm, 4.6 × 100 mm) were also screened using the same previous mobile phase composition of LC-MS. Similar results were obtained indicating the superiority of the Kinetex PFP column. Other parameters were then tweaked to optimize the separation. These parameters were injection volume (5–50 μL), flow rate (0.3–0.7 mL/min), and different elution gradients. Electrospray ionization was operated in the positive mode (ESI+), and parameters such as desolvation temperature (300, 400, and 500°C), cone voltage (30, 35, and 40V), and collision energy (10, 15, and 20V) were also examined before the selection of the optimum settings. Finally, 50 μL of commercial sample extracts were run at a flow rate of 0.4 mL/min using a gradient elution mixture of water and methanol both containing 0.1% formic acid for a total run of 7 min. Multiple reaction monitoring (MRM) with two transitions and a selected ion recording (SIR) method were employed to monitor NDMA and DMF, respectively. Ion transitions with a precursor to product ions of m/z = 75.05 ⟶ 58.05 (qualifier) and m/z = 75.05 ⟶ 43.0 (quantifier) were used for the NDMA analysis [51, 52]. A product ion with m/z = 74.06 was used for the determination of DMF. By solely fragmenting the NDMA precursor ion, the DMF ions that were interfering with the NDMA ions could significantly be reduced. The developed LC-MS/MS method was simple, specific, and sensitive to trace concentrations of NDMA as low as 1 ng/mL. Similarly, the LC-MS/MS method showed good linearity with a coefficient of determination (*r*^2^) ≥ 0.994 in the concentration range of 1–10 ng/mL. Regression analysis results showed that the predicted residual values of NDMA were similar to the actual values and were randomly scattered around the horizontal line, indicating that the linear model is a good fit to the data and repeatability over the selected range (Figure 4). The accuracy of the LC-MS/MS method expressed as the percent of recovery was close to 100% for all concentrations, showing that the developed method was reliable. The mean percent recoveries of NDMA from the QC samples at 1, 3, 5, and 10 ng/mL were 100% ± 1.7%, 99.0% ± 3.9%, 99.7% ± 4.2%, and 100.1% ± 0.7%, respectively. Besides, the mean NDMA recovery from PM samples with spiked 1 and 10 ng of NDMA was 97.6% ± 14.4% and 93.5% ± 11.5%, respectively. Further, the precision of the method was evaluated with respect to % RSD of the QC samples, which was found to be ≤ 8.0%, indicating that the method was precise in quantifying NDMA. The presence of 100 ng/mL of DMF did not significantly affect the retrieved NDMA peaks. The recovery of NDMA at 1, 5, and 10 ng/mL in the presence of 100 ng/mL of DMF was 117.2% ± 0.5%, 97.7% ± 1.6%, and 93.6% ± 0.8%, respectively.  Table 4 summarizes the values of method validation.

### 3.3. Optimization of NDMA Extraction

The importance of extraction techniques in analytical procedures to avoid the loss or the in situ formation of impurities, matrix interference, and ion suppression has been highlighted in the literature [53, 54]. Careful extraction methods should be applied with consideration to the nature of excipients, and the results should be carefully interpreted case-by-case, a guidance that is not reasonably feasible given the confidentiality on formulation compositions [38]. During the extraction work, we noticed that different studies used a concentration of either 100 mg of powder or API per mL of the extraction solvent [16, 43]. This implied that this ratio might be a golden threshold to minimize interference from excipients without excessive dilution of the sample. Therefore, a series of extraction solvents were screened including water, ether, cyclohexane, ethanol, DCM, methanol, and other hydroalcoholic mixtures (30%, 50%, 70%, and 90% methanol in water) by adding 10 mL of each solvent to a 1000 mg PM spiked with 100 ng/mL of NDMA, vortexing for 5 min followed by lateral shaking at 100 rpm and at 25°C temperature for 1 h. Samples were then centrifuged at 10,000 rpm for 30 min. The clear supernatant was then injected for analysis. Ethanol resulted in low intensity and poorly resolved NDMA peaks, while DCM was found to interfere with NDMA peaks. Cyclohexane and ether were difficult to handle and had very strong smell owing to their high volatility. They did not show any superiority in extraction under these conditions and thus were excluded. Among the other solvents tested, water and 50% methanol showed superior extraction power. Hence, 50% methanol was chosen as the extraction solvent for the commercial sample initial screening. The direct injection of the clear supernatant of the commercial sample extract showed significant amounts of NDMA in the tablets (M1; 29 ± 1.9 ng/tab, M2; 105 ± 10.2 ng/tab, M3; 54 ± 2.5 ng/tab, M4; 24 ± 3.7 ng/tab, and M5; 23 ± 1.9 ng/tab). Considering the sensitivity differences between both equipment, tablets containing the threshold concentrations (96 ng per 2550 mg max daily dose) or higher should be detectable in LC-MS (namely, M2). Surprisingly, only the DMF peak was observed, but there was no sign of NDMA peak upon running the same M2 sample through the LC-MS. This indicated the possible suppression of ionization due to the residual soluble excipients. At this point, there was no way to confirm the lack of interference from these residual excipients in LC-MS/MS measurements as well. Therefore, it was concluded that further cleaning of extracted samples is highly recommended to confirm the results. An indicator for success was set to achieving a relative matching of results between the two methods in commercial samples as that achieved in pure standard solutions.

**Figure 5:**
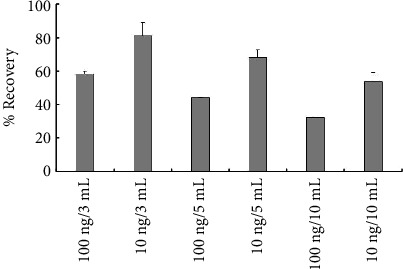
The recovery percentage of NDMA spiked into different volumes of water (3, 5, and 10 mL) and extracted by 1 mL of DCM (*n* = 3).

To address this issue, a double liquid–liquid extraction technique was pursued. In brief, the extraction was performed in two consecutive stages. In the first stage, the aqueous extract was equilibrated with an optimum volume of an organic layer for an adequate amount of time to extract the impurities from the dosage form. Water was selected as the aqueous phase over the 50% methanol in water to minimize the excipient solubilization, and DCM was selected as the organic phase due to its ease of handling and relative safety over ether and cyclohexane as well as its extraction efficiency [55]. The optimum volume of DCM, as well as the time required for equilibration, was studied prior to analyzing the commercial samples. A series of 3, 5, and 10 mL of water were spiked with low (10 ng/3–10 mL) and high (100 ng/3–10 mL) concentrations of NDMA and were then extracted by 1 mL of DCM. The tubes were vortexed for 2 min and then kept sealed on a lateral shaker for different periods of time (0.5–48 h) for comparison of extraction efficiency. The results identified an optimum DCM: water ratio of 1:3; thus, 3 mL of DCM should be used for a 10 mL volume of water (Figure 5). Although in clean samples, 1 h was sufficient to extract the NDMA efficiently, a 48 h period was selected for further experiments to ensure better partitioning of NDMA to the organic layer in the presence of excipients [43]. In the second stage, the organic layer was collected quantitatively in clean tubes and water was added. The mixture was placed in a water bath under nitrogen gas pressure to evaporate the organic layer only. This step was essential to eliminate the interference of DCM with NDMA due to coelution. Preliminary data showed that the evaporation of the DCM layer prior to reconstitution with water resulted in the coevaporation of NDMA (0% recovery), while the continuous addition of a thin layer of water during the drying process helped in complete re-extraction of the impurity (ca. 100% recovery). Thus, drying was confirmed to impose no negative effects on NDMA recovery as long as the water layer was continuously preserved [56]. Ultimately, all samples were adjusted to the same volume using water. The presence of formic acid in water showed better results in LC-MS, which could be explained by its enhancing effect on ionization (NDMA recovery was 69.9 ± 9.4% and 87.6 ± 2.2% from DCM spiked with 100 ng/mL and dried in the presence of water only and water with 2% formic acid, respectively). A final centrifugation was carried out as a precautionary step to ensure a clear supernatant for injection. This extraction protocol resulted in similar reproducible recovery % from both methods.

### 3.4. Analysis of Commercial Samples

**Figure 6:**
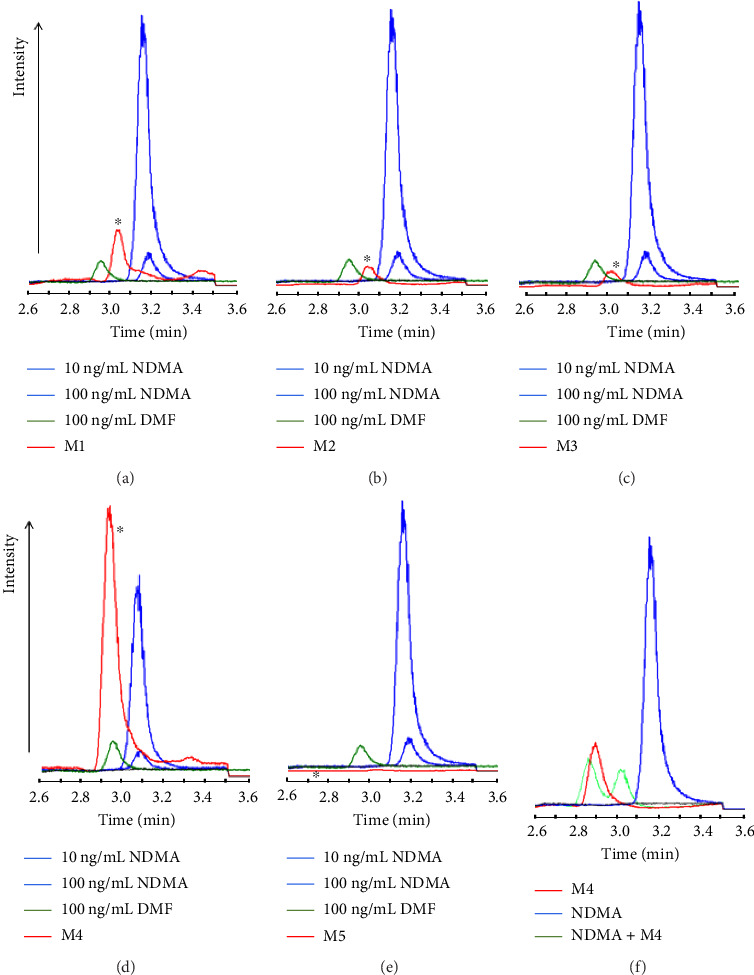
The analysis of five metformin commercial products for NDMA and DMF impurities. The five products were selected from a long list of products claimed to have appreciable amounts of NDMA. The chromatograms of commercial products (m/z = 75): (a) M1, (b) M2, (c) M3, (d) M4, and (e) M5, and (f) chromatograms of commercial product M4 before and after spiking with 100 ng/mL of NDMA (m/z = 75). The asterisk indicates the sample line. All samples are shown in comparison to 100 ng/mL of DMF and 10 and 100 ng/mL of NDMA standards (*n* = 3).

Five commercial metformin tablets were selected from a list of commercial products suspected to contain NDMA in various amounts.  Table 5 summarizes their properties. The tablets were extracted using the double liquid–liquid extraction technique with the aim to screen for the presence of NDMA and DMF as well as to quantify the NDMA amounts. For NDMA, the LC-MS analysis indicated that NDMA was not quantifiable in all the commercial products. When the samples were injected into the LC-MS/MS, all the observed peaks indicated minimal presence of NDMA augmenting the results of the single-stage MS. The maximum NDMA level was observed in M2 at 12 ± 0.2 ng/tablet (Figure 6). It is thus comprehensible that these trace amounts of NDMA were below or close to the LOQ. On the other hand, all tablets contained DMF in appreciable concentrations (Figures 6(a), 6(b), 6(c), 6(d), and 6(e)). Some tablets showed higher contents of DMF than others among the same set of replicates. In LC-MS, the range was roughly from 50 ng to 300.5 ng/tablet, while in LC-MS/MS, the DMF content ranged from 137.4 to 653.5 ng/tablet owing to the higher sensitivity of the latter. In both methods, the estimated concentrations of DMF were less than the permissible daily exposure (PDE) of 8.8 mg/day according to the ICH guideline (“International Council for Harmonization of Technical Requirements for Pharmaceuticals for Human Use. Impurities: Guideline for residual solvents Q3C(R6)”, 2016). The difference in DMF amounts detected by both methods might be attributed to the matrix effects in different detection modes and equipment sensitivities. To further confirm the ability of LC-MS to discriminate between DMF and NDMA simultaneously in commercial samples, M4 was spiked with 100 ng/mL of NDMA. Both peaks appeared in the chromatogram despite the change in the retention behavior, namely, the RT and peak areas, as shown in (Figure 6(f)). Both methods were in agreement that the tablets had varying amounts of DMF lower than its PDE, and the amounts of NDMA were below the acceptable intake (AI) of 96 ng/day as recommended by the FDA and other regulatory agencies (Table 6). Moreover, both methods were successful to eliminate the artifact caused by DMF in estimating the NDMA content.

## 4. Conclusion

Based on this study, we suggest the use of the LC-MS method as a tool for screening DMF simultaneously with NDMA in the drugs among the cohort of concern. Since the method is also capable of quantifying NDMA when present in alerting levels, it can be used to flag certain products for further sophisticated analysis using the sensitive tandem MS method with the same consumables (column and mobile phases). The use of this complementary protocol should save the money and effort in routine analysis of commercial products through their shelf life. It will also help ensure that recall efforts are focused on the appropriate products. LC-MS is a relatively cheap and easy-to-use analytical tool possessed by many laboratories. Moreover, the suggested analytical protocol provides a reciprocal verification procedures when test results from both methods match. It is expected that minor modifications might be required with different products of other drugs in the cohort of concern regarding NDMA impurity and those modifications should be tackled in future studies. In conclusion, two new analytical methods using LC-MS and LC-MS/MS were developed. Both methods showed good linearity (*r*^2^ = 0.971 & 0.994, respectively) and overall precision of < 8%. The methods were used to analyze five commercial metformin products suspected to have appreciable amounts of NDMA. Following optimization of extraction techniques and analytical procedures, the results of both methods were in harmony, confirming that the tested batches had NDMA traces below the FDA limit (AI = 96 ng/day). The methods successfully separated DMF simultaneously from NDMA, showing that the tablets tested contained appreciable amounts of DMF, yet, below its current permissible level (PDE 8.8 mg/day). The two methods with their strong discriminative power to NDMA and DMF eliminate the coelution artifacts of the two impurities and the potential for overestimation of NDMA. The two methods can be used in a complementary protocol for general screening and further qualification of flagged products. Both methods in combination should offer a reciprocal validation protocol before a concern is identified or a recall is issued. Finally, in this study, all the tested tablets met the safety criteria currently set by regulatory agencies.

## Figures and Tables

**Table 1:** Differential settings of UPLC-MS.

Probe temp. (^o^C)	400
Capillary voltage (V)	+1, −0.8
Cone voltage (V)	20 (for MS and SIR of mass 75) 5 (for SIR of mass 74)

**Table 2:** UHPLC-MS/MS settings.

Acquisition	MRM mode
Desolvation temp. (^o^C)	500
Desolvation gas flow (L/h)	1000
Cone gas flow (L/h)	0
Source temp. (^o^C)	150
Parent NDMA ion (m/z)	75.05
Daughter NDMA ions (m/z)	58.05, 43
Parent DMF ion (m/z)	74.06
Capillary voltage (kV)	0.8
Cone voltage (V)	35
Collision energy (V)	10
Dwell time (ms)	50

**Table 3:** Summary of method validation for UPLC-MS.

Parameter	Day 1	Day 2	Day 3	Mean ± SD
Linearity (correlation coefficient, *r*^2^)	0.995	0.974	0.968	0.971 ± 0.0
Slope	31219.83	31197.48	26372.18	29596.5 ± 2792.4
Confidence interval (CI 95%)	29596.8–32842.8	27381.6–35013.4	22745.3–29999.1	26574.6 ± 32618.4
*y*-Intercept	70599.3	9265.0	20893.8	33586.0 ± 32577.5
Confidence interval (CI 95%)	−25418.9–166617.5	−216488.2–235018.3	−193675.2–235462.8	−145194.1–212366.2

*Analytical range (10–100 ng/mL)*				
Calibrators	10	10	10	10
LOD (ng/mL)	—	—	—	1.9
LOQ (ng/mL)	—	—	—	5.6

*Accuracy (n = 6, % recovery)*				
10 ng/mL	94.4	78.2	75.3	82.6 ± 10.3
40 ng/mL	100.3	96.9	95.8	97.7 ± 2.3
70 ng/mL	103.5	102.8	107.0	104.4 ± 2.3
100 ng/mL	94.5	86.5	102.6	94.5 ± 8.1

*Precision (n = 6, % RSD)*				
10 ng/mL	10.5	23.6	3.1	12.4 ± 10.4
40 ng/mL	0.3	4.0	0.2	1.5 ± 2.2
70 ng/mL	4.3	1.6	0.0	2.0 ± 2.2
100 ng/mL	0.9	0.5	10.8	4.1 ± 5.8

**Table 4:** Summary of method validation for LC-MS/MS.

Parameter	Day 1	Day 2	Day 3	Mean ± SD
Linearity (correlation coefficient, *r*^2^)	0.995	0.989	0.997	0.994 ± 0.0
Slope	627.9	680.1	744.2	684.1 ± 58.3
Confidence interval (CI 95%)	565.8–689.9	580.1–780.1	690.4–798.0	612.1–756.0
*y*-Intercept	35.8	90.9	52.6	59.8 ± 28.2
Confidence interval (CI 95%)	−412.4–340.8	−515.9–697.7	−379.2–273.9	−435.9–437.5

*Analytical range (10–100 ng/mL)*				
Calibrators	6	6	6	6
LOD (ng/mL)	—	—	—	0.4
LOQ (ng/mL)	—	—	—	1

*Accuracy (n = 6, % recovery)*				
1 ng/mL	106.3	103.0	105.4	104.9 ± 1.7
3 ng/mL	103.4	96.2	97.4	99.0 ± 3.9
5 ng/mL	94.9	102.1	102.2	99.7 ± 4.2
10 ng/mL	100.9	99.8	99.7	100.1 ± 0.7

*Precision (n = 6, % RSD)*				
1 ng/mL	4.2	4.7	3.0	4.0 ± 0.9
3 ng/mL	2.3	4.1	5.2	3.9 ± 1.5
5 ng/mL	3.1	3.4	2.4	2.9 ± 0.5
10 ng/mL	2.5	2.5	3.2	2.7 ± 0.4

**Table 5:** Commercial products of metformin.

Product	M1	M2	M3	M4	M5
Shape	Round	Oblong	Oval	Oblong	Round
Color	White	White	White	White	Pink
Strength (mg/tablet)	500	500	500	500	500
Wt (mg) ± SD	524.2 ± 1.3	1045.4 ± 0.7	1062.2 ± 0.9	836.8 ± 0.8	1242.7 ± 2.3
Expiry	NA	NA	05/2025	NA	NA
Excipients	NA	NA	NA	NA	NA
Package	20 tablets/plastic tube	20 tablets/plastic tube	20 tablets/blister pack	20 tablets/plastic tube	15 tablets/plastic tube

**Table 6:** Summary of results.

Product	M1	M2	M3	M4	M5
LC-MS (water injection; ng/tablet)	0 ± 0.0	0 ± 0.0	0 ± 0.0	0 ± 0.0	0 ± 0.0
LC-MS/MS (water injection; ng/tablet)	0 ± 0.0	12 ± 0.2	8 ± 0.3	4 ± 1.1	4 ± 0.2

## Data Availability

The data that support the findings of this study are available from the corresponding author upon reasonable request.

## References

[1] Ghijs S., Wynendaele E., De Spiegeleer B. (2024). The Continuing Challenge of Drug Recalls: Insights From a Ten-Year FDA Data Analysis. *Journal of Pharmaceutical and Biomedical Analysis*.

[2] Patel R., Vhora A., Jain D. (2024). A Retrospective Regulatory Analysis of FDA Recalls Carried out by Pharmaceutical Companies From 2012 to 2023. *Drug Discovery Today*.

[3] AlSaeedy M., Al-Adhreai A., Oncu-Kaya E. M., Şener E. (2023). An Overview of Advances in the Chromatography of Drugs Impurity Profiling. *Critical Reviews in Analytical Chemistry*.

[4] Akkaraju H., Tatia R., Mane S. S., Khade A. B., Dengale S. J. (2023). A Comprehensive Review of Sources of Nitrosamine Contamination of Pharmaceutical Substances and Products. *Regulatory Toxicology and Pharmacology*.

[5] Bharate S. S. (2021). Critical Analysis of Drug Product Recalls due to Nitrosamine Impurities. *Journal of Medicinal Chemistry*.

[6] Charoo N. A., Dharani S., Khan M. A., Rahman Z. (2023). Nitroso Impurities in Drug Products: An Overview of Risk Assessment, Regulatory Milieu, and Control Strategy. *AAPS PharmSciTech*.

[7] Vikram H. P. R., Kumar T. P., Kumar G. (2024). Nitrosamines Crisis in Pharmaceuticals-Insights on Toxicological Implications, Root Causes and Risk Assessment: A Systematic Review. *Journal of Pharmaceutical Analysis*.

[8] Ponting D. J., Foster R. S. (2023). Drawing a Line: Where Might the Cohort of Concern End?. *Organic Process Research & Development*.

[9] Paustenbach D. J., Brown S. E., Heywood J. J., Donnell M. T., Eaton D. L. (2024). Risk Characterization of N-Nitrosodimethylamine in Pharmaceuticals. *Food and Chemical Toxicology*.

[10] EMA (2022). Questions and Answers for Marketing Authorisation Holders/applicants on the CHMP Opinion for the Article 5(3) No 726/2004 Referral on Nitrosamine Impurities in Human Medicinal Products.

[11] FDA (2021). Controls of Nitrosamine Impurities in Human Drugs: Guidance for Industry. https://www.fda.gov/regulatory-information/search-fda-guidance-documents/control-nitrosamine-impurities-human-drugs.

[12] FDA (2020). FDA Alerts Patients and Health Care Professionals to Nitrosamine Impurity Findings in Certain Metformin Extended-Release Products. https://www.fda.gov/drugs/drug-safety-and-availability/laboratory-tests-metformin.

[13] Manchuri K. M., Shaik M. A., Gopireddy V. S. R., Naziya S., Gogineni S. (2024). Analytical Methodologies to Detect N-Nitrosamine Impurities in Active Pharmaceutical Ingredients, Drug Products and Other Matrices. *Chemical Research in Toxicology*.

[14] Epa (2017). N-Nitroso-dimethylamine (NDMA). https://19january2021snapshot.epa.gov/sites/static/files/2017-10/documents/ndma_fact_sheet_update_9-15-17_508.pdf.

[15] FDA (2024). Control of Nitrosamine Impurities in Human Drugs Guidance for Industry. https://www.fda.gov/regulatory-information/search-fda-guidance-documents/control-nitrosamine-impurities-human-drugs.

[16] Dharani S., Mohamed E. M., Rahman Z., Khan M. A. (2024). Patient In-Use Stability Testing of FDA-Approved Metformin Combination Products for N-Nitrosamine Impurity. *AAPS PharmSciTech*.

[17] Miralles P., Chisvert A., Salvador A. (2018). Determination of N-Nitrosamines in Cosmetic Products by Vortex-Assisted Reversed-Phase Dispersive liquid-liquid Microextraction and Liquid Chromatography With Mass Spectrometry. *Journal of Separation Science*.

[18] Luo F., Liu Y., Xie Y., Hou W., Zhang L., Zhang Z. (2022). Simultaneous Determination of 13 Nitrosamine Impurities in Biological Medicines Using Salting-Out Liquid-Liquid Extraction Coupled With Liquid Chromatography Tandem Mass Spectrometry. *Journal of Pharmaceutical and Biomedical Analysis*.

[19] Géhin C., O’Neill N., Moore A., Harrison M., Holman S. W., Blom G. (2023). Dispersant-First Dispersive Liquid-Liquid Microextraction (DF-DLLME), a Novel Sample Preparation Procedure for NDMA Determination in Metformin Products. *J Pharm Sci*.

[20] Aggarwal P., Sharma G., Singh V., Dev R., Kumar A. (2024). Solid-Phase Extraction Followed by Gas chromatography-Mass Spectrometry for the Quantitative Analysis of Small Molecule N-Nitrosamine Impurities in Antitussive Syrups. *Journal of Chromatography A*.

[21] Planinšek Parfant T., Roškar R. (2025). A Comprehensive Approach for N-Nitrosamine Determination in Pharmaceuticals Using a Novel HILIC-Based Solid Phase Extraction and LC-HRMS. *Talanta*.

[22] Yamamoto E., Kan-No H., Tomita N., Ando D., Miyazaki T., Izutsu K. I. (2022). Isolation of N-Nitrosodimethylamine From Drug Substances Using Solid-Phase Extraction-Liquid Chromatography-Tandem Mass Spectrometry. *Journal of Pharmaceutical and Biomedical Analysis*.

[23] Lim H. H., Oh Y. S., Shin H. S. (2020). Determination of N-Nitrosodimethylamine and N-Nitrosomethylethylamine in Drug Substances and Products of Sartans, Metformin and Ranitidine by Precipitation and Solid Phase Extraction and Gas Chromatography-Tandem Mass Spectrometry. *Journal of Pharmaceutical and Biomedical Analysis*.

[24] Giménez-Campillo C., Pastor-Belda M., Campillo N., Hernández-Córdoba M., Viñas P. (2021). Development of a New Methodology for the Determination of N-Nitrosamines Impurities in Ranitidine Pharmaceuticals Using Microextraction and Gas Chromatography-Mass Spectrometry. *Talanta*.

[25] Ishizaki A., Ozawa K., Kataoka H. (2023). Simultaneous Analysis of Carcinogenic N-Nitrosamine Impurities in Metformin Tablets Using On-Line In-Tube Solid-Phase Microextraction Coupled With Liquid Chromatography-Tandem Mass Spectrometry. *Journal of Chromatography A*.

[26] Vogel M., Norwig J. (2022). Analysis of Genotoxic N-Nitrosamines in Active Pharmaceutical Ingredients and Market Authorized Products in Low Abundance by Means of Liquid Chromatography-Tandem Mass Spectrometry. *Journal of Pharmaceutical and Biomedical Analysis*.

[27] FDA (2020). M7(R2) Assessment and Control of DNA Reactive (Mutagenic) Impurities in Pharmaceuticals to Limit Potential Carcinogenic Risk. Questions and Answers. https://www.fda.gov/drugs/drug-safety-and-availability/laboratory-tests-metformin.

[28] https://www.valisure.com/wp-content/uploads/Valisure-FDA-Citizen-Petition-on-Metformin-v3.9.pdf.

[29] Yang J., Marzan T. A., Ye W., Sommers C. D., Rodriguez J. D., Keire D. A. (2020). A Cautionary Tale: Quantitative LC-HRMS Analytical Procedures for the Analysis of N-Nitrosodimethylamine in Metformin. *The AAPS Journal*.

[30] Muzart J. (2009). N,N-Dimethylformamide: Much More Than a Solvent. *Tetrahedron*.

[31] Patent (2015). A Kind of Method That Two-Component Solvent Prepares High-Purity High-Yield Metformin Hydrochloride. https://patents.google.com/patent/CN104829495B/en.

[32] International Council for Harmonisation of Technical Requirements for Pharmaceuticals for Human Use (2016). Impurities: Guideline for Residual Solvents Q3C(R6).

[33] Li M. J., Zeng T. (2019). The Deleterious Effects of N,N-Dimethylformamide on Liver: A Mini-Review. *Chemico-Biological Interactions*.

[34] National Center for Biotechnology Information (2025). PubChem Compound Summary for CID 91403745, CID 91403745. https://pubchem.ncbi.nlm.nih.gov/compound/N_N-dimethylformamide.

[35] Ogbede J. U., Giaever G., Nislow C. (2021). A Genome-Wide Portrait of Pervasive Drug Contaminants. *Scientific Reports*.

[36] Aldawsari F. S., Alshehry Y. M., Alghamdi T. S. (2021). N-Nitrosodimethylamine (NDMA) Contamination of Ranitidine Products: A Review of Recent Findings. *Journal of Food and Drug Analysis*.

[37] Commission Regulation (Eu) 2021/2030 (2021). Amending Annex XVII to Regulation (EC) No 1907/2006 of the European Parliament and of the Council Concerning the Registration, Evaluation, Authorisation and Restriction of Chemicals (REACH) as Regards N,N-Dimethylformamide. https://eur-lex.europa.eu/eli/reg/2021/2030/oj.

[38] Sherwood J., Albericio F., de la Torre B. G. (2024). N,N-Dimethyl Formamide European Restriction Demands Solvent Substitution in Research and Development. *ChemSusChem*.

[39] Shi S., Li K., Peng J. (2022). Chemical Characterization of Extracts of Leaves of Kadsua Coccinea (Lem.) A.C. Sm. by UHPLC-Q-Exactive Orbitrap Mass Spectrometry and Assessment of Their Antioxidant and Anti-inflammatory Activities. *Biomedicine & Pharmacotherapy*.

[40] Metformin Global Market Report (2025). Method for Preparation of High Purity and High Yield Metformin Hydrochloride by two-component Solvent. https://www.thebusinessresearchcompany.com/report/metformin-global-market-report.

[41] https://www.definitivehc.com/resources/healthcare-insights/top-outpatient-prescription-medications.

[42] Zhou Z., Luo G., Li C. (2023). Metformin Induces M2 Polarization via AMPK/PGC-1α/PPAR-γ Pathway to Improve Peripheral Nerve Regeneration. *American Journal of Translational Research*.

[43] Dharani S., Mohamed E. M., Khuroo T. (2022). In-Use Stability Assessment of FDA Approved Metformin Immediate Release and Extended Release Products for N-Nitrosodimethylamine and Dissolution Quality Attributes. *International Journal of Pharmaceutics*.

[44] Snyder L. R. (2016). The Hydrophobic-Subtraction Model for Reversed-Phase Liquid Chromatography: A Reprise. *LCGC North America*.

[45] Stoll D. R. S. (2020). Selectivity in Reversed-Phase Liquid Chromatography: 20 Years of the Hydrophobic Subtraction Model. *LCGC North America*.

[46] Shimadzu Technical Support (2025). *Key Differences in the Use of Methanol and Acetonitrile*.

[47] Jadeja S., Kupcik R., Fabrik I., Sklenářová H., Lenčo J. (2023). A Stationary Phase With a Positively Charged Surface Allows for Minimizing Formic Acid Concentration in the Mobile Phase, Enhancing Electrospray Ionization in LC-MS Proteomic Experiments. *Analyst*.

[48] Wu Z., Gao W., Phelps M. A., Wu D., Miller D. D., Dalton J. T. (2004). Favorable Effects of Weak Acids on Negative-Ion Electrospray Ionization Mass Spectrometry. *Analytical Chemistry*.

[49] Epa (2000). N,N-Dimethylformamide-Technical Fact Sheet. https://www.epa.gov/sites/default/files/2016-09/documents/n-n-dimethylformamide.pdf.

[50] Sheng Y.-h., Jue W., Jiang Y.-P. (2024). Comparison of Metabolomics Peak-Picking Parameter Optimization Algorithms Based on Chromatographic Peak Shape. *Chinese Journal of Analytical Chemistry*.

[51] Jireš J., Kalášek S., Gibala P. (2021). Insight into the Formation of N-Nitrosodimethylamine in Metformin Products. *Journal of Pharmacy Biomedicine Analytical*.

[52] Zhao Y. Y., Liu X., Boyd J. M., Qin F., Li J., Li X. F. (2009). Identification of N-nitrosamines in Treated Drinking Water Using Nanoelectrospray Ionization high-field Asymmetric Waveform Ion Mobility Spectrometry With Quadrupole Time-of-Flight Mass Spectrometry. *Journal of Chromatographic Science*.

[53] Fritzsche M., Blom G., Keitel J. (2022). NDMA Analytics in Metformin Products: Comparison of Methods and Pitfalls. *European Journal of Pharmaceutical Sciences*.

[54] Williams M. L., Olomukoro A. A., Emmons R. V., Godage N. H., Gionfriddo E. (2023). Matrix Effects Demystified: Strategies for Resolving Challenges in Analytical Separations of Complex Samples. *Journal of Separation Science*.

[55] Parr M. K., Joseph J. F. (2019). NDMA Impurity in Valsartan and Other Pharmaceutical Products: Analytical Methods for the Determination of N-Nitrosamines. *Journal of Pharmaceutical and Biomedical Analysis*.

[56] Schlingemann J., Boucley C., Hickert S. (2022). Avoiding N-Nitrosodimethylamine Formation in Metformin Pharmaceuticals by Limiting Dimethylamine and Nitrite. *International Journal of Pharmaceutics*.

